# A Second Verdict against an Apothecary for a Mistake in Putting up Veratrum Viride, Instead of Valerian, Thereby, as Alleged, Causing Death

**Published:** 1867-04

**Authors:** J. R. Lothrop


ART. II—*A Second Verdict against an Apothecary for a mistake in putting up Veratrum Viride, instead of Valerian, thereby, as alleged, causing death.* By J. R. Lothrop, M. D.

This case, brought into court for the arbitrament of a jury, a second time, that ill-starred remedy, veratrum viride. In the case about to be considered the main facts were these. A woman, near 60 years of age, residing at Silver Creek, took two doses of Tilden's fluid extract of veratrum viride, each dose being somewhere from 25 to 40 drops, at about six hours interval. Vomiting followed the taking of the medicine, and continued more or less for twenty-seven days after, when it was ended by death.

The mistake occurred in this wise. A phial which had been previously often filled with fluid extract of valerian—a remedy habitually taken by the woman—was brought to the shop of Mr. Haven to be again filled with valerian. Mr. Haven took down from the usual place on the shelf, as he supposed, the bottle which contained the medicine asked for, and filled the phial from it. He did not carefully read the label of the bottle from which he poured the medicine, but looking at it hastily, and observing that the first letter was "v," and taking for granted that all the shelf bottles were properly placed, a doubt of his giving the right medicine never entered his mind. The fact was, however, that there had been a wrong placing of the shelf bottles, so that, that containing veratrum viride was in the place of that containing valerian, and hence the mistake.

The first dose, (25 to 40 drops) was taken near midnight, and shortly after caused vomiting, followed by quiet for some time, but not marked prostration or distress. The second dose varying not essentially from the first in quantity, was given early in the morning, and was immediately followed by vomiting and distress, and many of the described symptoms consequent upon a large dose of veratrum viride. Up to the time of taking the second dose, there was no suspicion of a mistake in the medicine, the effects following, however, soon made it evident. From the time of the second dose till death took place, there was more or less vomiting, and of course inability to retain food in the stomach; and, as alleged, death by exhaustion. Such were the facts presented by the prosecution, though not in full, admitted by the defence.

A post-mortem examination of the body was made, at which were present Drs. Ward, Horton and Cheesman of Silver Creek, Dr. Harrison of Sinclairville, Dr. Strong of Westfield, Dr. Rogers of Dunkirk, and Dr. Johnson of Buffalo. The autopsy revealed traces of former pericarditis, but no detected structural change in the heart itself, or its valves. Upon this point there was no disagreement. The stomach was contracted, *all* admitted. As to thickening of its coats there was not an entire agreement—some thinking it thickened and others not. Upon the point of traces of inflammation there was a difference of opinion, but it seems to have been given out as the united statement, (whether rightly or not,) that the appearances of the stomach were sufficiently indicative of gastritis—in other words, that there were softening and discoloration of the mucous membrane, and in all the coats evident congestion. I say it was given out as the opinion of all, but in justice I must add, that several of the gentlemen said that neither at the time, nor afterwards, did they have or express such an opinion. On the contrary, the stomach appeared to them abnormal only in size. All agreed that the intestines were normal, and the kidneys likewise, though they were not taken out, only lifted and briefly examined. The brain was not examined. The liver was normal, as were also the organs of the pelvis. Thus as far as the autopsy was concerned nothing appeared as a reasonable cause for death, aside from the gastric abnormities, and as to those there was a difference of opinion. I should say that Dr. Rogers was inclined to think the pericardiac læsion serious.

Now, in fairness, I think it must be said that those medical gentlemen who did, in sincerity, believe they saw in the stomach the læsions of gastritis, had some warrant for supposing that all thought so. For in the first place those who differed did not positively state their dissent at the time, and in the second place the autopsy was not very thoroughly made, that is, it was stopped short of a complete examination, as if a definite cause of death had been found, and further search was useless. In fact these gentlemen went so far as to say, that they expressed a desire to have the autopsy continued to the point of satisfaction to all, either positively or negatively, and that all declared themselves satisfied, viz: as to the cause of death being found in the lesions of the stomach.

But, on the other hand, this is denied; several gentlemen leaving, entirely unsatisfied as to the finding of a cause for death, and thinking the whole indefinite and vague. Now, why was it? In the first place the examination was made at night, with defective light, and in a hurry, so that some of the gentlemen might be in time for the midnight train. In the second place, they were summoned to be present at a case of poisoning, and were therefore less prepared to look for læsions in any other organ than the stomach; and prepared even to find them absent, inasmuch as poisons do not always leave traces. They took for granted the fact stated, that it was a death by poisoning, and therefore were not at the time aware of the importance of searching for any other cause of death. Thus it happened that they did not openly dissent from, though they did not expressly assent to, the opinions entertained by others, viz: that gastritis had left traces sufficient to make it chargeable with the death.

This seems to account most naturally for the evident lack of careful investigation. The death was from poison, and it might or might not have left traces of local action. Those who went there expecting to meet with it would be more likely to find it. But to the others the apparent fact of death by poisoning was the most important one. Some of them undoubtedly felt as one of them said upon the stand, that they were playing the part of "second fiddle," though that was not a proper feeling if it hindered a thorough examination. It is probable that all did not anticipate that the case might come into court, and therefore did not fully realize the importance of a careful observation. And here it may be said, without prejudice and in all candor, that it never should have been brought to trial, for there was really nothing in the case, as the testimony will show, although the sympathy of the jury led them to award $300 damages. It may be further said, it never could have been brought into court had not the relatives been encouraged by medical opinions, if not advice. As it was, though the verdict was against the apothecary, no great end of justice or equity was promoted, and it led to an unpleasant agitation in the locality where it occurred, a conflict of professional opinion never very edifying to the public, some exraordinary swearing on the stand, and withal a sore disappointment to rather high-raised hopes of great damages.

But I will now detail the case as it appeared in court. The trial was had at Mayville, at the Special Term of the Supreme Court. Judge Marvin presiding.

The case as presented by the testimony of the relatives—a son, a son-in-law, and two daughters of the deceased—was substantially this. A woman of near 60 years of age, in good health, able to be about and work, having no ailment to attract attention, except occasional palpitation, for which she had been advised to take valerian—in other words of previous good health—took by mistake a medicine which caused constant vomiting for a month, when she died from its effects, as they believed. No suspicion was created by their testimony that they had ever been anxious about her health or thought her an invalid. They had, however, sought the advice of Dr. Ward of Silver Creek, at various times before the taking of the wrong medicine; had not a great while before sought the advice, in consultation, of Dr. Rogers of Dunkirk, and soon after that of Dr. Harrison of Sinclairville. Yet they were able to say on the stand they had had no anxiety about their mother's health. As before said, they substantially testified that their mother then and previously in good health, took a poison which in the end wrought her death.

Dr. Ward, her attending physician, by his testimony did not materially alter this aspect of the case. He had seen her some times in a professional and sometimes in a friendly way, at various times before the mistake, but had not thought her seriously ill, in fact he did not appear to feel any anxiety about her, thinking her trouble a functional, nervous disturbance. I do not remember that he said anything that would give the impression that he agreed with Drs. Harrison and Rogers in their more serious view of the case. In regard to the effects of the medicine, he gave a decided opinion that the vomiting, consequent exhaustion and finally death were to be attributed to it. In other words veratrum viride acted as a gastric irritant, causing inflammation of the stomach and the other consequences. He thought the stomach showed traces of gastritis.

Dr. Harrison, in the main, agreed with Dr. Ward as to the effects of the medicine and cause of death. He thought she would not have died if she had not taken the medicine. He thought the stomach showed traces of gastritis. Upon these points it is not necessary to state his testimony in detail. But upon one point Dr. Harrison's testimony was very important, and a model of can-dor and clearness, viz: upon the point of health previous to the taking of the medicine. He did not like Dr. Ward give the idea that the deceased was merely affected with functional disturbances, but he confessed that he thought her ailments serious, and might very properly be a source of anxiety to her children. He had known her in former years as a woman of sound health, and when called to her in consultation with Dr. Ward, was struck with the change which had taken place in her appearance. She had, to him, the look of serious illness. He found upon examination a quick and irregular pulse, and an œdematous state of the lower limbs. Examination by auscultation appeared to him to reveal the presence of organic disease of the heart. Under such circumstances death was probable at any time. The autopsy did not confirm his expectations as to structural change in the heart.

Dr. Rogers had also, from his examination when called in consultation, formed the opinion that organic disease of the heart existed, and that death might occur at any time. To him, also, the autopsy was a disappointment as to structural læsion of the heart, yet upon reflection he was not satisfied it did not exist, but escaped observation in the hurry of a night post-mortem examination. As to the condition of the stomach, he did not think there were traces of disease sufficient to account for death. As to the cause of death, he said he thought the pericardiac lesion and its disturbing influence upon the heart was the chief agent in bringing it about. He did not undertake to say that the veratrum viride might not have accelerated death, but he denied its having the principal or even a large share in the fatal result.

Dr. Strong was present at the autopsy, and was clearly of the opinion that the stomach exhibited decided traces of gastritis; he saw softening, and at points destruction of its mucous coats, and he said "it was the most congested stomach I ever saw." He thought the medicine caused death. When asked if he had ever heard of a death caused by veratrum viride, he said he believed Dr. Beck spoke of its having been fatal. A reply wanting in fairness, for the remark of Dr. Beck warrants no assertion that veratrum viride has caused death. Dr. Beck merely says, "*It is said* that dangerous and fatal effects have followed its use." A statement very far from a positive assertion that it had in any case proved fatal.

I have given above with an endeavor at accuracy of purport, such parts of the testimony of the medical witnesses called by the prosecution as seems to me most material. That of the attending and two consulting physicians is of the most interest. It will not escape notice that the case presented by the testimony of the consulting physicians was very different from that testified to by the attending physician. It is difficult to reconcile the latter with the former on any theory of fairness or intelligent observation. In regard to the case presented by the relatives, it is sufficient to say that it is only entitled to be thought candid, either from great confidence in the opinion of the attending, or from little confidence in the opinions of the consulting physicians.

On the part of the defense Drs. Johnson, Horton, Cheeseman and the writer were called to testify. One part of the examination of each related to the general question of the action of veratrum viride, which being well known, and having been fully discussed in a former number of the *Journal*, (case of Farnell vs. Greene, tried at Lockport,) need not now be gone into. It is conceded that as yet there is no fatal case directly chargeable upon veratrum viride. The point in this case was its properties as an *irritant;* and all the medical witnesses for the prosecution had each something to say about its "irritation." The medical witnesses for the defense denied to it any special power to act by irritation, in such a degree as to cause gastritis, and thus remotely cause death. It is enough to pass this point over with the above notice, at present, and further on give the question more extended examination. As to the question, therefore, whether veratrum viride could by its properties give rise to gastritis, all the gentlemen connected with the defense denied it. As to the fact of there being traces of gastritis in the stomach, the three of them present at the autopsy thought they did not exist.

Dr. Johnson removed and laid the stomach open, and upon careful examination of it, saw nothing abnormal about it, but its size. It was smaller than the average. As to the heart, he did not find clear evidence of structural change in its substance, but did observe traces of a former pericarditis. Drs. Horton and Cheeseman agreed with Dr. Johnson mainly as to the condition of the heart and stomach. Dr. Cheeseman had seen the deceased during her life, say a week or two before her death. He was attending one of the daughters of deceased, and being frequently in the house, saw her during her illness. He never found her vomiting; but he noticed delirium.

The theory of the prosecution was, that the veratrum viride acting as an irritant caused inflammation of the stomach, which by keeping up prolonged vomiting and consequent rejection of all food, ended in death. The death, therefore, was primarily chargeable upon the medicine.

The theory of the defense was, that any such action of veratrum viride, especially in the small quantity taken, was so unlike what is known of it, that it was more probable that some other cause acted coincidently and was the principal one. It might be sympathetic with cardiac disturbance or disease, or some obscure, undetected affection of the brain or kidneys, (Bright's disease.) Moreover, the deceased was in feeble health, the victim of some organic disease, as evidenced by dropsy of limbs, cardiac disturbance and failing strength.

It is possible, though I am not aware, that this view was presented, that veratrum viride, whose *modus operandi* is through the nervous system, might operate is some unexplained way by its powerful and depressing impression upon the nervous system to keep up vomiting and thus destroy life. But this would be inconsistent with the theory that it caused vomiting by its local influence in creating a lesion of the stomach; unless we answer that prolonged vomiting of itself would bring about those changes which were testified to by several physicians. But though it is difficult to say what is possible; from what is known, it is altogether probable that the influence of veratrum viride is temporary, and therefore not likely to continue. And as this theory would exclude local irritation, we should be compelled to assume that the vomiting was kept up by habit, as it were, the prime impression on the nervous system having worn off. These considerations seem to reduce the prosecution to the necessity of assuming as a cause of vomiting and hence death, a local irritant action.

Now we may ask, could the patient have died of acute gastritis? In the first place admitting that there were traces of gastritis, there were none of inflammation of the duodenum, or of the tongue, pharynx, or œsophagus. These should have been found, or were probable, if the lesion of the stomach was due to an irritant action. Contraction and congestion are both results of gastritis, but the first may happen from mere want of distention by food, in prolonged vomiting, and the second is very common in cardiac lesions. Moreover, gastritis from any such cause is rapid usually, running its course in *days*, not *weeks*. It did not appear in evidence, but it was observed by some of the physicians at the autopsy that the body was not much wasted—a fact inconsistent with gastritis and persistent vomiting for twenty-seven days. And lastly, vomiting itself, persistent vomiting is not, alone absolutely indicative of affection of the stomach. Affection of the brain, as for instance chronic meningitis, with no symptoms referable to the brain, may be attended with persistent vomiting. The same is true, in a measure, of the later stages of Bright's disease.

On the other hand, on the side of the defense, there are many circumstances to show that such a disturbance as vomiting was altogether probable. These have been mentioned. There were symptoms present which pointed pretty directly to that chronic affection of the kidneys known as Bright's disease, and there is fair ground for the assumption that it needed but a careful testing of the urine during life, to have been in possession of this indubitable evidence of such affection. The defense, moreover, by the testimony of competent observers denied the fact, that the autopsy revealed traces of gastritis. It is only necessary to account for the declared fact that vomiting began with the giving of the medicine and persisted, thereby seeming clearly connected with it as a cause. The only reply to this that can be made, admitting the fact, is that it was coincident. Now, lame as this may appear to a non-professional man, to a medical man stranger coincidences are not uncommon. Assume, and it can be seen what ground there is for the assumption, that the deceased had a disease in which vomiting was an event sooner or later to be expected, and there is nothing improbable in its setting in at, or about the time, the unfortunate mistake was committed.

It has been said above that the case ought never to have come to trial, and probably would not had not the family been encouraged by medical opinions, if not advice. This is recalled to attention, for the purpose chiefly of making the statement more full; but also, expressly to disclaim any reflections upon the motives of the medical men connected as witnesses with the prosecution. They acted, we believe, from the best motives; thinking it highly important to public safety, as well as to spare medicine itself from reproach, that mistakes of such serious nature should be followed by some penalties. We may believe that, by an indirect influence upon public confidence, they felt such things were injurious to the profession itself.

Still, it appears that, whatever they might have hoped from the example, there was not enough of the case to have got any aid from them. The connection between the taking of the medicine and the death does not appear to have been clear enough to put them in the relation of cause and effect. At any rate it appears to be not warranted by experience hitherto, with the remedy. Whatever may be thought of this by others, medical men should be careful of any such deduction, and without medical testimony uch a case would come to nought.

Several reasons occur why such cases should get no encouragement from medical men. In the first place, they are sure to get a verdict from a jury, even with slight medical support, not only beyond, but sometimes in opposition to what is just. As they generally are brought for damages, the amount obtained is always larger than it should be, sometimes excessive. Juries will mostly believe, that a powerful medicine is the cause of any bad effects which may follow the giving of it, if a medical man can be found to say that they were *possible.* Therefore we repeat, they should be careful of giving a positive opinion where there is doubt. A mere possibility should not be a basis of opinion; even quite strong probabilities should not be allowed too much weight.

Secondly, they lead to conflict opinion before juries, and hence diminish the value of medical testimony. On many subjects there is room for difference. It is easy to see how Doctors may differ, and from no mere love of contradiction. But in any cases involving error of judgment or mistakes, all doubt should help the defense, and positive opinions should be expressed only when they are founded upon certainty or such a weight of probability as almost to amount to it. The case above related affected an apothecary, not a physician; but success in such a case, renders suits against physicians themselves, for mistakes, much more likely to be brought. Trials which result in a very just punishment to quacks even, for most ignorant mal-practice, in the end injure the profession, by stimulating all those who think they have been badly treated, or who really have experienced results, confessedly bad, but the best possible under the circumstances, to the hope of getting a pecuniary satisfaction. But if fair meaning men become connected with a case in which, even, there is much better ground for positive opinion than there was in this, they should strive to avoid becoming *ex parte*, and by hasty inferences, unfair or extravagant replies, seek to gain advantage for the side on which they appear.

In the legal view of the case, there was less of it than in the medical even. By law, a person having suffered by gross negligence, can recover for the personal injury and pain even, brought upon him by it. But in case of death by negligence, the heirs of the deceased can recover only for the pecuniary loss brought upon them by the death; as for instance, loss of service, or actual expense in consequence. Sympathy for the pain inflicted upon deceased, or for the affliction caused to the heirs, is to be ruled out of the question. In the case above, all they were entitled to recover, was their actual money damage, first for the loss of services of an aged and feeble mother, and secondly the expenses of sickness and burial, supposing the connection between the mistake and the death indubitable. It can be seen what motive may have influenced the relatives to make out a case of fair health, before the mistake happened. We should not expect such considerations would have any influence with the family physician.

The point in this case was slightly unlike that in the Lockport case. In that, veratrum viride was accused of acting as a violent intestinal irritant, no evidence being brought forward to prove that it affected the stomach in any unusual way. In this case, on the other hand, veratrum viride was charged with acting as a violent gastric irritant, but neither in life exhibiting, or after death leaving, signs of any intestinal irritation. In the one case, it expended by selection its force upon the intestinal mucous membrane, in the other upon the gastric. In both, the question of its action as an irritant was brought forward. It again brings up the question whether it is to be classed as an irritant poison. It is clear that it is not known to have caused death. It has been given in large doses without very permanent bad effects. This would hardly have happened if it was an active irritant. Writers speak of its being acrid, some say very acrid, but this is not enough to place it in the class of irritant poisons. A corrosive poison might act alone on the stomach, an irritant would not be likely to. Up to this time, there is not much support in medical opinion or experience, for the idea that veratrum viride is to be ranked among irritant poisons.

